# Neurobiology Research on Neurodegenerative Disorders

**DOI:** 10.3390/brainsci14111121

**Published:** 2024-11-04

**Authors:** Grażyna Lietzau

**Affiliations:** Division of Anatomy and Neurobiology, Faculty of Medicine, Medical University of Gdańsk, Dębinki 1, 80-211 Gdańsk, Poland; grazyna.lietzau@gumed.edu.pl

The aim of the following Special Issue was to call for research in the field of neurodegenerative disorders (NDDs). Despite the growing interest in this field over the past few decades, the unquestionable progress in understanding the mechanisms underlying NDDs and numerous attempts to find effective therapies, many questions remain unanswered. Moreover, since it is not possible to reverse the progressive degeneration of neurons in various regions of the central nervous system (CNS), NDDs are still considered incurable. As such, further research is warranted. The scope of this Special Issue covers articles on diagnostics, mechanisms underlying NDDs, and new or modified therapeutic strategies.

Despite the decreasing trend in dementia observed recently in North America and Europe [1], given population growth and ageing trends, the number of people affected by dementia is expected to increase worldwide [1]. This makes dementia a rapidly growing global public health problem. To meet this challenge, new studies are being designed and attempts are being made to develop new treatments. Numerous studies have been conducted to test the effectiveness of various substances in treating dementia, including Alzheimer’s disease (AD), using animal models of the disease. Various doses and routes of administration have been tested in different studies. The results of two such studies are presented in this Special Issue. Lu et al. investigated mechanisms at the base of the neuroprotective action of Apelin-13 (Contribution 1), the endogenous ligand of the apelin receptor (APJ) involved in processes in the CNS such as inflammation, oxidative stress, apoptosis, and autophagy. The researchers used a streptozotocin (STZ)-induced model of AD, in which STZ (3 mg/kg) was injected into the lateral ventricles of C57BL/6J mice. The above model is a widely used model of sporadic AD. The results showed that the intranasal administration of Apelin-13 (1 mg/kg) improved cognitive function in AD mice. The functional outcome correlated with the enhancement of synaptic plasticity and the attenuation of oxidative stress. The authors conclude that intranasal administration of Apelin-13 may prove to be a promising therapeutic strategy for neurodegenerative diseases such as AD.

Kim et al. studied the effect of gut microbiome composition on the pathology and progression of AD (Contribution 2). They report that orally administered probiotics, *Bifidobacterium lactis*, *Levilactobacillus brevis*, and *Limosilactobacillus fermentum*, improve spatial and recognition memory and have neuroprotective effects in 5XFAD mice. These transgenic mice are a commonly used model of AD-like amyloid pathology with a relatively early onset and aggressive age-dependent progression. Based on the improvement of cognitive function and the observed reduction in amyloid-β accumulation, decreased microglial activation, and ameliorated increased tau phosphorylation in 5xFAD mice, the authors suggest that probiotics may prove to be an effective neuroprotective intervention in AD. Although animal models of NDDs have certain limitations, they represent a useful tool for studying disease mechanisms and exploring the neuroprotective potential of new or modified therapeutic strategies. The endogenous substances used in the two aforementioned studies affected well-known mechanisms involved in AD: oxidative stress and inflammation. Importantly, they improved cognitive function. However, there is a long way to go to determine their effectiveness in patients with AD.

Parkinson’s disease (PD) is the second most common progressive NDD [2]. In addition to the characteristic motor symptoms (tremor, bradykinesia, rigidity, and postural instability), patients with PD also experience non-motor symptoms such as psychological dysfunctions (depression, anxiety, and apathy) and cognitive impairment/dementia [3]. Most patients also develop olfactory dysfunction and sleep disorders, including REM sleep behavior disorder (RBD). Although in recent years more attention has been paid to the non-motor symptoms of PD, many issues related to the role of olfaction in the development and progression of NDDs, as well as the interrelationships between different non-motor symptoms, still await explanation. In their study, Solla et al. investigated the association between olfactory impairment and RBD in PD (Contribution 3). The aim of the study was to determine the role of olfactory dysfunction and other factors (i.e., age at onset, sex, cognitive abilities, and motor symptoms) as potential predictors of higher scores on the RBD screening questionnaire. The authors opined that because new disease-modifying drugs will have the greatest chance of being effective in the early stages of NDDs, identifying olfactory dysfunction and RBD as early as possible may be useful both in clinical practice and in clinical trials of potentially neuroprotective treatments. The main finding of the study was the demonstration of a strong association between olfactory impairment and the studied sleeping disorder. The authors reported that the presence of more severe olfactory impairment strongly correlated with a more symptomatic expression of RBD.

PD is a complex disorder affecting many processes in the CNS. The risk of this NDD increases with age, with the peak incidence occurring in the eighth decade of life. Stress hormones, such as cortisol, may contribute to neurodegeneration. Stress-induced mitochondrial dysfunction, neuroinflammation, and oxidative stress can directly damage dopaminergic neurons in the substantia nigra or increase the vulnerability of these cells to other damaging factors. Consequently, stress exacerbates the symptoms of PD [4]. In their review, Luthra et al. summarize the current knowledge on the role of two hormones, cortisol and Klotho, in PD (Contribution 4). While cortisol is a well-known stress hormone, Klotho is an aging-suppressor protein that has been shown to protect against stress-induced damage, particularly oxidative stress and inflammation, which are often exacerbated by chronic high levels of cortisol. The authors conclude that aging and stress are associated with increased cortisol and decreased Klotho levels, whereas exercise and certain genetic variants lead to decreased cortisol response and increased Klotho levels in PD individuals. Together, they influence the clinical presentation of PD. Elucidating the interacting pathways and the role of antagonistic factors may allow for a better understanding of the complexity of PD and potentially enable the development of more effective therapies.

Another interesting topic included in this Special Issue is addressed by Bohnen and colleagues (Contribution 5). In their study, they investigated the relationship between the CNS regional availability of GABA_A_R benzodiazepine binding sites and motor impairments in PD. A total of 11 male patients with PD underwent [^11^C]flumazenil GABA_A_R benzodiazepine binding site and [^11^C]dihydrotetrabenazine vesicular monoamine transporter type-2 (VMAT2) PET imaging and clinical assessment. The results indicated that decreased availability of GABA_A_R benzodiazepine binding sites in the thalamus, reflecting increased GABAergic activity, correlated with increased axial motor impairments in PD, independently of the degree of nigrostriatal neurodegeneration. The findings suggest that GABA_A_R benzodiazepine binding site allosteric modulator drugs could be effective in managing axial motor impairments in PD. Since there is still no cure for PD and available therapies such as levodopa/carbidopa, deep brain stimulation, and rehabilitation only alleviate symptoms, it is crucial to search for new treatment options.

Although amyotrophic lateral sclerosis (ALS) is a rare disease (prevalence in Europe: ~10–15/100,000; worldwide annual incidence: ~1.9/100,000), it represents the most aggressive NDD [5]. Selective and progressive degeneration of motor neurons both in the spinal cord and brain usually leads to death within 2–5 years of diagnosis. The projected increase in the number of individuals with ALS between 2015 and 2040 is 69% (prognoses for 10 geographical regions) [6]. Such alarming statistics necessitate taking action to better understand this NDD and design effective treatments or at least limit the effects of ALS in order to prolong patients’ lives and improve their quality of life. In their study, Lin et al. investigated the spatiotemporal expression of suppressor of cytokine signaling-3 (SOCS3) in the brainstem and spinal cord of ALS mice (Contribution 6). SOCS3 is a regulator of neuroinflammation that acts primarily by inhibiting the JAK-STAT signaling pathway and controlling the activity of microglia and the production of pro-inflammatory cytokines. The results of in vivo and in vitro studies showed a negative regulatory effect of SOCS3 on neuronal survival and axon regeneration [7], and the authors hypothesized that it participates in ALS progression. As a model of ALS, the B6.Cg-Tg (SOD1*G93A)1Gur/J mouse has been used in a number of studies. The SOD1-G93A transgenic mouse is one of the commonly used ALS models in preclinical studies, involving neurodegeneration of both upper and lower motor neurons. The results of a study showed upregulation of SOCS3 in the pre-Bötzinger complex of the brainstem and in the ventral horn of cervical and lumbar segments of the spinal cord, accompanied by increased astrogliosis and microglia activation as well as neuronal loss in these areas. These results correlated with the progression of ALS from the pre-symptomatic to early symptomatic stage. The authors conclude that SOCS3 is involved in the neuroinflammation-associated non-cell-autonomous pathway in the course of ALS and that it may be a potential therapeutic target for balancing the neuroinflammatory response to regulate ALS progression. Despite recent criticism that the use of transgenic mice has not yielded rapid advances in the prevention and treatment of ALS, they remain a useful tool in studying the pathogenic processes in ALS and tracking disease progression.

In the next article included in this Special Issue, Suthar et al. explore the relationship between superoxide dismutase 1 (SOD1) and ALS (Contribution 7). SOD1 is an antioxidant enzyme that protects cells from free radical-mediated damage. Mutations in the SOD1 gene result in cellular stress and the development of ALS. Furthermore, the number of SOD1 variants that cause ALS is increasing (Contribution 7). Suthar et al. used a bioinformatics approach (including ingenuity pathway analysis) to analyze the signaling pathways, regulatory functions, and network molecules of SOD1. They reported that SOD1-mediated toxicity is related to swelling and oxidative stress and the key signaling pathways involving this enzyme are degradation of superoxide radicals, apelin adipocyte, NRF2-mediated oxidative stress response, ALS, and sirtuin signaling (Contribution 7). Further analysis facilitated the identification of specific molecules in the SOD1-ALS pathway. Modern bioinformatics tools enable the analysis of large data sets, and the obtained results may indicate further directions for in vivo research on ALS.

The third ALS-related article included in this Special Issue is a review by Schirò et al., in which the authors discuss cellular clonotypic immunity in ALS (Contribution 8). This NDD is primarily known for the progressive degeneration of motor neurons; however, increasing evidence suggests that immune mechanisms, including adaptive immunity, might play a role in its development or progression. The role of clonotypic T cells in ALS is complex. From one perspective, regulatory T cells (Tregs), specific T cell clones that are responsible for maintaining immune tolerance and controlling excessive immune responses, may play a protective role by reducing inflammation and potentially slowing disease progression. Conversely, the clonal expansion of cytotoxic T cells (CD8+ T cells) contributes to motor neuron damage by promoting inflammation and attacking neurons or glial cells in the CNS, exacerbating neurodegeneration. A high percentage of CD8+ cells in ALS patients correlates with a higher risk of death [8]. These and other aspects of immune cell function in ALS are summarized in the review. The authors conclude that the prognosis of ALS may be influenced by the balance between CD4+ and CD8+ cells and between Treg and Teff cells (effector T lymphocytes) (Contribution 8). Attempts have been made to treat ALS with immunomodulatory agents. Studies involving larger cohorts and a better understanding of the immune mechanisms at the onset and progression of ALS may enable the development of more effective treatment strategies.

Skogholt’s disease (SD) is a disease characterized by white matter lesions in the brain and myelin damage in the peripheral nerves, with high concentrations of copper and iron in the CSF, first described in the 1980s in southeastern Norway by a local physician, Jon Skogholt [9]. This rare disorder is only present in a familial line in Hedmark County, Norway. Aspli et al. performed a neurochemical analysis of plasma and cerebrospinal fluid and morphometric segmentation of the brain using MRI (Contribution 9). The results showed increased concentrations of Aβ_1–42_, Aβ_1–40_, Aβ_x–38_, Aβ_x–40_, phosphorylated and total tau protein, GFAP, PDGFRβ, and β-trace protein in the CSF, in addition to decreased white matter volume and choroid plexus volume and increased gray matter volume and cortical thickness in 11 patients with SD compared to the control group. Further research involving larger cohorts is warranted to better characterize this NDD and to find effective treatment methods.

Early diagnosis of cognitive decline provides better prospects for halting its progression. Neurocognitive disorders (NCDs) have many etiologies such as AD, vascular disease, Lewy body disease, frontotemporal disorders, PD, Huntington’s disease, multiple sclerosis, traumatic brain injury, prion disease, and others. Current challenges in the diagnosis of progressive NCDs are presented in the review by Abbatantuono et al. (Contribution 10). The authors conducted searches on online databases (PubMed and Scopus) to investigate the neurocognitive stages characterized by signs and clinical manifestations preceding the onset of mild and/or major NCDs. They discuss the diagnostic criteria and stadial models for NCDs in the context of their usefulness in primary and secondary care. The review focuses on the following stages of neurocognitive decline: the preclinical stage, the transitional stage, the prodromal or mild stage, and major NCD. The authors conclude that the identification and monitoring of individuals at all stages from preclinical to overt dementia are essential for optimizing clinical efforts against neurocognitive decline.

Another type of neurocognitive disorder is normal pressure hydrocephalus in which the accumulation of CSF in the brain ventricles leads to a range of symptoms, including cognitive decline. Idiopathic normal pressure hydrocephalus (iNPH) is the most common form of hydrocephalus in the adult population and may accompany NDDs such as AD and PD [10]. Porru et al. analyzed levels of oxysterols before and after ventriculoperitoneal shunt surgery and reported a significantly lower level of these oxygenated derivatives of cholesterol in iNPH patients before surgery and an increase in 24-OH and 7HOCA levels following surgery (Contribution 11). Based on the obtained results, the scientists conclude that oxysterols present in the CSF may find potential use as biomarkers in the diagnosis and management of iNPH.

The projections of an increase in the incidence of NDDs worldwide in the coming years are alarming and the situation requires urgent attention. Multidirectional actions combining prevention, early diagnosis, and therapies that halt disease progression, in addition to the search for neuroregenerative therapies, should prove effective in the fight against NDDs in the long term. Identifying new biomarkers, better understanding the role of the immune system and hormonal regulation in NDDs, and the use of bioinformatics tools to discover connections between different pathways and processes in the course of NDDs are just some of the research approaches presented in this Special Issue. Different approaches and increased efforts focused on various aspects of NDDs should shed more light on their development and course and, consequently, lead to the development of more effective treatments.
